# Can psychiatric childhood disorders be due to inborn errors of metabolism?

**DOI:** 10.1007/s00787-016-0908-4

**Published:** 2016-09-30

**Authors:** A. Simons, F. Eyskens, I. Glazemakers, D. van West

**Affiliations:** 1 Centre of Heriditary Metabolic Diseases Antwerp (CEMA), University Hospital of Antwerp (UZA), Wilrijkstraat, 2650 Edegem, Belgium; 2 Collaborative Antwerp Psychiatric Research Institute (CAPRI) Youth, Antwerp, Belgium; 3 University of Brussels, Brussels, Belgium; 4 University Child and Adolescent Psychiatry Antwerp, Lindendreef 1, 2020 Antwerp, Belgium; 5 University of Antwerp (CAPRI), Universiteitsplein 1, 2610 Wilrijk, Belgium

**Keywords:** Metabolic disorders, Child psychiatric disorder, ASD, ADHD, Learning disorder, Psychosis, Eating disorder

## Abstract

Many patients who visit a centre for hereditary metabolic diseases remarkably also suffer from a child psychiatric disorder. Those child psychiatric disorders may be the first sign or manifestation of an underlying metabolic disorder. Lack of knowledge of metabolic disorders in child psychiatry may lead to diagnoses being missed. Patients therefore are also at risk for not accessing efficacious treatment and proper counselling. To search the literature for the co-occurrence of child psychiatric disorders, such as ADHD, autism, psychosis, learning disorders and eating disorders and metabolic disorders. A search of the literature was conducted by performing a broad search on PubMed, using the terms “ADHD and metabolic disorders”, “autism and metabolic disorders”, “psychosis and metabolic disorders”, “learning disorders and metabolic disorders”, and “eating disorders and metabolic disorders”. Based on inclusion criteria (concerning a clear psychiatric disorder and concerning a metabolic disorder) 4441 titles and 249 abstracts were screened and resulted in 71 relevant articles. This thorough literature search provides child and adolescent psychiatrists with an overview of metabolic disorders associated with child psychiatric symptoms, their main characteristics and recommendations for further investigations.

## Introduction

Although a lot of research has already been done about organic causes of child psychiatric disorders, few of them focus on metabolic disorders as a possible cause of a child psychiatric disorder. Many children who visit our centre for hereditary metabolic diseases suffer from a child psychiatric disorder [1]. Metabolic disorders cover a variety of diseases in which there is an accumulation of toxic and/or complex compounds or energy problems within the cells due to enzymatic defects or other protein dysfunction (e.g., transporter defects).

Sometimes the psychiatric symptoms occur before irreversible neurological lesions. A number of metabolic disorders give rise to a major psychiatric disorder. These metabolic disorders can result in neuropsychiatric illness either through disruption of late neurodevelopmental processes, or via chronic or acute disruption of excitatory/inhibitory or monoaminergic neurotransmitter systems. This disruption to metabolic processes can lead to gross neurodevelopmental disruption with seizures and coma, or to mild disruption with intermittent and/or subtle cognitive, behavioural disturbance and psychiatric illness, such as psychosis [2]. To prevent or decrease mortality, morbidity and disabilities associated with metabolic diseases as much as possible, it is important to detect the metabolic disease as early as possible. For this reason, it is important that child and adolescent psychiatrists are aware of possible underlying metabolic disease in child psychiatric problems. This is of great importance because specific treatment may be available to prevent metabolic decompensation and further progression of disease can be avoided. In addition, many of these conditions have important implications for genetic counselling. This article gives an overview of the literature on co-occurring metabolic disorders and child psychiatry disorders and attempts to give child psychiatrists some recommendations on when to screen for metabolic disorders.

## Method

We searched PubMed for articles published between 1 January 1980 and 31 December 2013, using the search terms: “ADHD and metabolic disorders”, “autism and metabolic disorders”, “psychosis and metabolic disorders”, “learning disorders and metabolic disorders”, and “eating disorders and metabolic disorders”. Concerning psychosis, we included articles about visual auditory or visual hallucinations, paranoid delusions, interpretative thoughts and schizophrenia. Articles were selected according the following criteria: (1) articles were written in English, (2) the article concerned a clear psychiatric disorder according the DSM-criteria and concerned a metabolic disorder, and (3) the article was not about a metabolic syndrome.

**Fig. 1:**
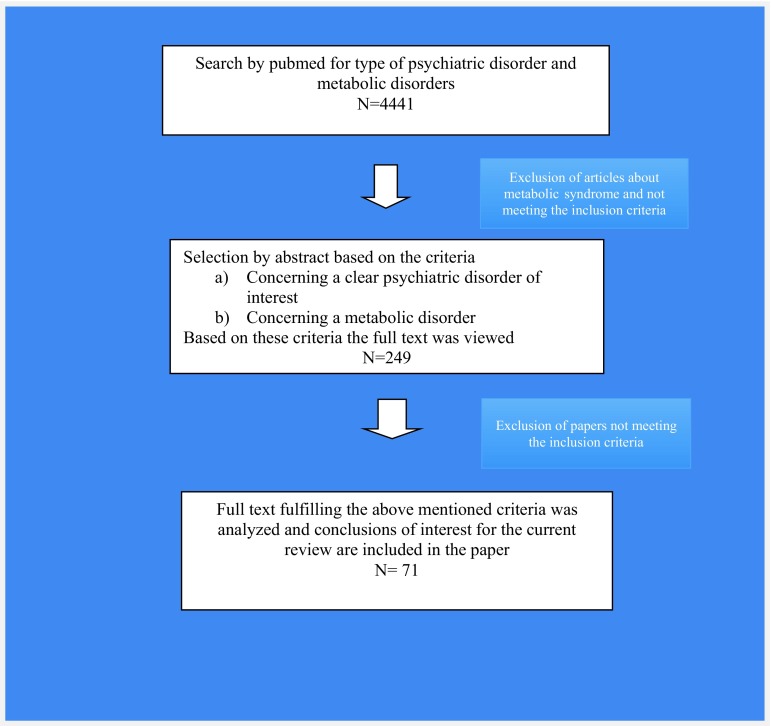
Flow diagram showing process of literature search

Our search resulted in 4441 initial hits, after screening titles and abstracts for inclusion and exclusion criteria, we studied the remaining 249 articles and concluded that only 71 were actually relevant (and not concerning the metabolic syndrome). Figure 1 shows the flow diagram that was used for all psychiatric disorders in the literature search; Table 1 specifies the search results for each psychiatric disorder separately.

**Table 1:** Results of PubMed search by type of psychiatric disorder

Type of psychiatric disorder	Number of titles found	Number of abstracts viewed	Numbers of articles included for review
ADHD	316	34	10
Autism	559	101	19
Eating disorders	1513	18	5
Learning disorders	726	49	17
Psychosis	1327	47	20
Total (including review)	4441	249	71

Psychiatric disorders such a depression and anxiety disorders were not included in the search because based on clinical experience and earlier research [1], we expect these disorders rather to be a consequence of dealing with the diagnosis of and life with a metabolic disease than that they share a common underlying disruption.

## Literature search

There is a lack of review articles on the subject. Only three reviews on psychiatric symptoms and metabolic disorders were found.

One by Sedel et al. [3] proposed a classification of metabolic diseases into three groups according to the type of psychiatric signs at onset. Group 1 represents psychiatric emergencies, namely acute and recurrent attacks of confusion and behavioural changes, sometimes misdiagnosed as acute psychosis. This includes urea cycle defects, homocysteine remethylation defects and porphyrias. Group 2 includes diseases with chronic psychiatric symptoms arising in adolescence or adulthood. These psychiatric symptoms can be recurrent psychotic attacks, chronic delusion or disorganized behaviour, and behavioural and personality changes. Among these diseases are homocystinurias, Wilson disease, adrenoleukodystrophy and some lysosomal storage disorders. Group 3 is characterized by mild mental retardation and late-onset behavioural or personality changes. This group includes homocystinurias, cerebrotendinous xanthomatosis, nonketotic hyperglycinemia, monoamine oxidase A deficiency, succinic semialdehyde dehydrogenase deficiency, creatine transporter defect, and alpha-/beta-mannosidosis. In this paper, a diagnostic strategy to guide metabolic investigations in a patient with atypical psychiatric signs is proposed.

Another article by Estrov et al. [4] reviewed four metabolic diseases: phenylketonuria (PKU), Wilson disease, acute intermittent porphyria (AIP) and metachromatic leukodystrophy (MLD). Early treated PKU can exhibit depressed mood, anxiety (esp agoraphobia) and psychosocial difficulties. Wilson disease can present with personality changes, depressive episodes, cognitive dysfunction and psychosis. AIP is often associated with symptoms of anxiety, depression, psychosis and altered mental status as psychiatric manifestations. MLD frequently presents with psychosis followed by intellectual deterioration.

In 2013, Walterfang et al. [2] wrote a review on the neuropsychiatry of inborn errors of metabolism. In this article, following metabolic disorders are also associated with psychiatric symptoms: metachromatic leukodystrophy, GM2 gangliosidosis, adrenoleukodystrophy, Niemann–Pick type C disease, cerebrotendinous xanthomatosis, neuronal ceroid lipofuscinosis, alpha-mannosidosis, Fabry disease, AIP, maple syrup urine disease, urea cycle disorders, disorders of homocysteine metabolism and PKU. Remarkably, there is an increase in reports the latest years about mitochondrial dysfunctioning and several neurodevelopment disorders, such as ASD, learning disorders, ADHD, schizophrenia and mood disorders [5, 6]. A mitochondrial dysfunction leads to an energy problem and neural synapses are areas of high energy consumption.

### Autism spectrum disorders

About the link of autism spectrum disorder (ASD) and metabolic disorders four review articles were found [7–9]. In these four reviews, concerning ASD and metabolic disorders, similar findings are reported. Known metabolic disorders in autism are phenylketonuria, disorders in purine metabolism (such as adenosine deaminase deficiency, adenylosuccinate lyase deficiency, dihydropyrimidine dehydrogenase and dihydropyrimidinase deficiencies), organic acidurias (such as propionic academia, 3-methylcrotonyl-CoA carboxylase deficiency and pyridoxine dependency), disorders of branched-chain amino acids creatine deficiency, biotinidase deficiency, cerebral folate deficiency, succinic semialdehyde dehydrogenase deficiency, Smith–Lemli–Opitz syndrome (SLOS), late infantile ceroid lipofuscinosis, histidinemia, Sanfilippo disease, glucose 6-phosphate dehydrogenase deficiency, urea cycle disorders, X-linked ichthyosis, and mitochondrial disorders. In most of these disorders, there is also mental or psychomotor retardation.

In addition, case reports and some small studies about these diseases were found [10–14]. Benvenuto [15] names PKU, adenylosuccinase deficit, SLOS, creatine deficiency syndromes and mitochondrial disorders as possible causes of syndromic autism. Case reports of autism in association with acute intermittent porphyria [16], propionic academia [17] and L-2-hydroxyglutaric aciduria [18] were found. Kaluzna-Czaplinska [19] focusses on the role of homocysteine in autism. A high level of blood and urinary homocysteine is associated with pathophysiology of ASD and may serve as a diagnostic tool for the detection of nutrient deficiencies (folate, vitamin B12) in autistic children. The last years several studies were done suggesting a disturbance of mitochondrial energy production as an underlying pathophysiological mechanism in autism [20–22]. Frye [6] states that children with ASD and a mitochondrial disorder appear to have a higher prevalence of medical disorders such as gastrointestinal problems, seizures and gross motor delay. An interesting study was done in Greece [23] where they screened 187 children with confirmed features of ASD for the presence of inborn errors of metabolism. Their data provide evidence for a new biomarker (3-OH-IVA) and novel treatment approaches in ASD patient. Biotin supplementation or institution of a ketogenic diet resulted in mild to significant clinical improvement in the autistic features. Table 2 summarizes, based on the literature, when ASD is combined with specific other psychiatric, neurodevelopmental or somatic problems with metabolic diseases can be an underlying cause.

**Table 2:** Possible underlying metabolic diseases in ASD

Associated signs and symptoms	Possible underlying metabolic disease	Other signs and symptoms of the metabolic disease	Onset of the metabolic disease
Mental retardation and behavioural problems (such as aggressive behaviour, hyperactivity, impulsivity, agitation)	Untreated PKU Disorders in purines metabolism Lesh–Nyhan Succinic semialdehyde dehydrogenase deficiency Smith–Lemli–Opitz syndrome Mucopolysaccharidosis type II (Hunter) X-linked ichthyosis	Seizures, eczema Seizures, spastic paraplegia, self-mutilation Seizures, hypotonia, ataxia, intermittent lethargy, speech delay Feeding difficulties in infancy, muscle hypotonia, facial dysmorphism, expressive language disorders, excessive screaming in early childhood, genital abnormalities in the male Retinitis pigmentosa, chronic diarrhoea, regression, sleep problems Scaly skin on the scalp, trunk and limbs (ichthyosis)	Neonatal First years After 3 months After infancy During first year From birth
Regression	Cerebral folate deficiency Mucopolysaccharidosis type II (Hunter)	Deceleration of head growth, psychomotor retardation, cerebellar ataxia, dyskinesias, seizures, apnea, megaloblastic anaemia Cfr supra	1 -12 months Cfr supra
Seizures or epilepsy	Untreated PKU Disorders in purines metabolism Dihydropyrimidine dehydrogenase deficiency Dihydropyrimidinase Deficiency Cerebral Creatine deficiency Biotinidase deficiency Biotinidase or Late-onset carboxylase deficiency Succinic semialdehyde dehydrogenase deficiency Late infantile ceroid lipofuscinosis Urea cycle disorders Hyperammonaemia Acute intermittent porphyria (AIP) L-2-hydroxyglutaric aciduria Mitochondrial disorders OXPHOS defects	Cfr supra Cfr supra Psychomotor retardation, growth retardation, failure to thrive, microcephaly, ocular abnormalities, self-mutilation, muscle weakness, haemolytic anaemia, asymptomatic patients Extrapyramidal signs, speech delay Ataxia, hypotonia, organic acidemia, alopecia, skin rash, visual and hearing loss Cfr supra Developmental delay, speech acquisition delay, myoclonus, ataxia, choreiform movement, visual loss, dementia Hepatomegaly, abnormal hair, hepatic fibrosis, intellectual and developmental disabilities, learning disabilities, confusion, delusion, tone change, reflex abnormalities, vomiting, headache, changing food habits Anxiety, restlessness, insomnia, psychosis, aggressive and impulsive behaviour, catatonia, intestinal problems, dark urine, skin lesions, acute peripheral neuropathy (paralysis of diaphragm) acroparesthesia Developmental retardation, macrocephaly, cerebellar ataxia Central nervous system dysfunction, behavioural disturbance, cognitive impairment, motor disturbances, muscle hypotonia and exercise intolerance	Cfr supra Cfr supra During first year 3 months –2 year 1–12 months Cfr supra After 2 years From birth OTC is a X-linked disorder with episodic clinical presentation in females Adolescence to adulthood Especially in women Childhood From infancy to adulthood
Speech or language impairment	Cerebral Creatine deficiency Smith–Lemli–Opitz syndrome Late infantile ceroid lipofuscinosis Histidinemia Propionic acidemia	Cfr supra Cfr supra Cfr supra Mental retardation, asymptomatic patients Behavioural problems, hyperammonaemia, metabolic acidosis	Cfr supra Cfr supra Cfr supra Infancy to childhood Neonatal and infancy

### Attention deficit hyperactivity disorder

The literature search found several studies concerning the prevalence of attention deficit hyperactivity disorder (ADHD) in persons with a metabolic disorder. Knerr [11] studied a population of 33 subjects with succinic semialdehyde dehydrogenase deficiency of which 28 % showed behavioural problems such as attention deficit and hyperactivity. In addition, autistic features were found in these patients. The patients in this population also have psychomotor retardation. In a population of 25 boys with X-linked ichthyosis [14], 40 % fulfilled DSM-IV criteria for a diagnosis of ADHD. ADHD in children with PKU is well documented in several papers. Antshel [24, 25] stated that prenatal exposure to elevated levels of phenylalanine is associated with a higher likelihood of expressing hyperactive/impulsive symptoms and postnatal exposure is associated with a higher likelihood of expressing inattentive symptoms. Arnold [26] performed a study in 38 children with PKU and found that 26 % of this children use stimulant medication for attentional dysfunction, which is significantly higher than in an age- and sex-matched control group. Case reports of ADHD in a child with a metabolic disorder were found for 3-methylcrotonyl-CoA carboxylase deficiency [27], argininosuccinate lyase deficiency [28] and succinyl-CoA: 3-oxoacid CoA transferase deficiency [29].

Wijburg et al. [30] stated that mucopolysaccharidosis III (Sanfilippo disease), which is characterized by progressive cognitive decline and severe hyperactivity, is often misdiagnosed as an idiopathic developmental delay, ADHD or ASS. ADHD is also linked to mitochondrial disorders [5]. An overview of the metabolic disorders associated with ADHD is summarized in Table 3.

**Table 3:** Possible underlying metabolic diseases in ADHD

Associated symptoms and signs	Possible underlying metabolic disease	Other symptoms and signs of the metabolic disease	Onset of the metabolic disease
Autism and mental retardation	Untreated PKU Succinic semialdehyde dehydrogenase deficiency X-linked ichthyosis Mitochondrial disorders	Mental retardation, seizures Hypotonia, speech delay, seizures, ataxia, psychomotor retardation, intermittent lethargy Scaly skin on the scalp, trunk and limbs (ichthyosis), mental retardation Central nervous system dysfunction, behavioural disturbance, cognitive impairment, motor disturbances, seizures, muscle hypotonia and exercise intolerance	Neonatal After 3 months From birth From infancy to adulthood
Speech delay	Succinic semialdehyde dehydrogenase deficiency Succinyl-CoA: 3 oxo acid CoA transferase (SCOT) deficiency Mucopolysaccharidosis Type III (Sanfilippo syndrome)	Cfr supra Ketoacidotic crises, persistent ketosis, lethargy, coma, speech and behavioural problems, sleep disorders, anxiety, hallucinations, aggressive behaviour, seizures Developmental or speech delay after a period of normal development, behaviour problems, hyperactivity, mild facial dysmorphism, frequent ear and respiratory infections, chronic diarrhoea	Cfr supra Infancy to adulthood Childhood
Hypotonia	Succinic semialdehyde dehydrogenase deficiency Nonketotic hyperglycaemia 3-methylcrotonyl-CoA carboxylase deficiency	Cfr supra History of infantile hypotonia and feeding difficulties, mental retardation, ADHD, outburst of aggressiveness, sexual impulsivity Neurological abnormalities and death in infancy, feeding difficulties, recurrent episodes of vomiting and diarrhoea, lethargy, secondary carnitine deficiency, asymptomatic patients	Cfr supra Infancy to adulthood Infancy to adulthood

### Learning disorders

Taking learning disorders into consideration, some reports are found showing a link between learning disorders and adrenoleukodystrophy [31, 32] and MLD [33], which is associated with nonverbal learning disability. Gordon [34] reported about glucose transporter type 1 deficiency as a preventable cause of severe learning difficulties. Children with PKU show lower IQ, slow information processing, reduced learning capacity, mild executive impairments, and educational difficulties [35]. Antshel [24, 25] also reports in a review that young adults with PKU are more likely to have academic difficulties than their non-PKU peers, especially in maths. This is due to ADHD but also to executive functioning deficits and processing speed deficits. Janzen [36] stated that individuals with PKU also have nonexecutive cognitive abilities, such as problems with information speed processing, fine motor control, and perception and visual-spatial abilities. In an American longitudinal observation with 108 individuals with urea cycle disorders, 35 % had learning disabilities [37]. Lichter [38] specified this for OTC-deficiency. Special emphasis in this article was made on the late-onset (partial) disease, who can present from infancy to adulthood. A hyperammonemic crisis can lead to a life-threatening event and neuropsychological complications, such as developmental delay, ADHD and executive function deficits. Potter [39] studied 43 children with galactosemia and a history of speech sound disorders. 56 % of the children with typical cognitive development and 88 % of the children with borderline-low cognitive development showed language impairments. The first group had more often an expressive language disorder, the second group more often a mixed receptive-expressive language disorder. Bahi-Buisson [40] examined 22 patients with hyperinsulinism-hyperammonaemia syndrome and found a learning disability in 17 patients. Case reports were found about the occurrence of learning disorders in glutaric aciduria type I [41] and generalised uridine diphosphate galactose-4-epimerase deficiency [42]. Learning difficulties are also reported in Niemann-Pick disease type C [43]. Patients with juvenile neuronal ceroid lipofuscinosis show learning delay and regression of acquired skills [44]. Brady [45] described two cases with mucopolysaccharidosis type IIIB presenting as children with behavioural issues and mild learning disabilities, and having a rapid cognitive decline in early adulthood (see Table 4 for an overview).

**Table 4:** Possible underlying metabolic disease in learning disorders

Associated signs and symptoms	Possible underlying metabolic disease	Other symptoms and signs of the metabolic disease	Onset of the metabolic disease
Neurodegeneration	Adrenoleukodystrophy (X-linked) Metachromatic leukodystrophy Glutaric aciduria type 1 Mucopolysaccharidosis Type III (Sanfilippo syndrome)	Variable phenotype, deterioration in school performance, dementia, vision loss, sensorineural hearing loss, brain white matter abnormalities on MRI, adrenal insufficiency Progressive neurodegeneration. Nonverbal learning disability syndrome, spasticity, brain white matter abnormalities, peripheral neuropathy Acute regression after an initial phase of (almost) normal development (acute encephalopathic crisis), severe dystonic-dyskinetic movement disorder, macrocephaly, MRI of the brain: fronto-temporal atrophy Developmental or speech delay after a period of normal development, behaviour problems, hyperactivity, mild facial dimorphism, frequent ear and respiratory infections, chronic diarrhoea	Childhood to adulthood Infancy to adulthood 6 months to 2 years Childhood
Seizures	Untreated PKU Glucose transporter type 1 deficiency Hyperinsulinism-Hyperammonaemia syndrome Urea cycle disorders Juvenile neuronal ceroid lipofuscinosis	Autism, ADHD, mental retardation, executive impairment Delayed development in infancy with acquired microcephaly (cerebral/cerebellar atrophy), ataxia Hypoglycaemia, weakness, shakiness, rapid pulse, confusion Hepatomegaly, abnormal hair, hepatic fibrosis, intellectual and developmental disabilities, learning disabilities, tone change, reflex abnormalities, vomiting Progressive deterioration of cognition, ataxia, spasticity, vision loss, learning delay, regression of acquired skills	Neonatal Neonatal Infancy From birth on Infancy to adulthood
Vomiting and/or diarrhoea	Galactosemia Urea cycle disorders Mucopolysaccharidosis Type III (Sanfilippo syndrome)	Lethargy, vomiting, diarrhoea, failure to thrive, jaundice, cataract, speech difficulties, learning disorders, tremor, ovarian failure, osteoporosis Cfr supra Cfr supra	Infancy to childhood Cfr supra Cfr supra

### Psychosis

Psychosis, what can be auditory or visual hallucinations, paranoid delusions, and interpretative thoughts, and can be a symptom of schizophrenia, is described in alpha-mannosidosis [46, 47] and also in other lysosomal storage diseases: late-onset Tay–Sachs disease [48] and Fabry disease [49, 50]. Psychosis is also seen in mitochondrial disorders, in particular, respiratory chain defects [5, 51, 52]. Wilson’s disease can present with psychosis, but also as personality and mood changes, depression, phobias, cognitive impairment, anxiety and compulsive and impulsive behaviour [53–55]. A clinical presentation of metachromatic leukodystrophy during adolescence and/or adulthood may be psychosis [56]. If a post pubertal patient presents with acute mental changes and hallucinations or psychosis, acute porphyria should be considered [57]. In addition, a link between a disturbed homocysteine metabolism and schizophrenia is described [58]. Psychosis is also described in Niemann-Pick type C [59, 60]. Also, behavioural disturbances occur in this disease (see Table 5 for an overview).

**Table 5:** Possible underlying metabolic diseases in psychosis

Associated signs and symptoms	Possible underlying metabolic disease	Other symptoms and signs of the metabolic disease	Onset of the metabolic disease
Seizures	Mitochondrial disorders Acute intermittent porphyria (AIP) Urea cycle disorders Methylenetetrahydrofolate reductase deficiency	Central nervous system dysfunction, behavioural disturbance, cognitive impairment, motor disturbances, autism Anxiety, restlessness, insomnia, neuropathy, psychosis, aggressive and impulsive behaviour, catatonia, intestinal problems, dark urine, skin lesions, epilepsy, acute peripheral neuropathy Hepatomegaly, abnormal hair, hepatic fibrosis, intellectual and developmental disabilities, learning disabilities, confusion, delusion, seizure disorders, muscle tone change, reflex abnormalities, vomiting, changing food habits, headache Mild or severe depending on the enzyme activity: encephalopathy, gait disturbance, paraparesis, arterial or venous thrombosis and strokes, neurocognitive impairment, feeding problems, spasticity	Infancy to adulthood Adolescence and adulthood Infancy to adulthood Infancy to adulthood
Mental retardation	Alpha- Beta-mannosidosis Niemann–Pick type C Homocystinuria	Immune deficiency, facial and skeletal abnormalities, hearing impairment, intellectual disability, progressive neurological signs, episodes of confusion and psychosis followed by a period of confusion, somnolence and asthenia Hepatosplenomegaly, cerebellar ataxia, dysarthria, vertical gaze palsy, cognitive difficulties, progressive neurological deterioration, psychotic symptoms, schizophrenia, behavioural disturbances (aggressiveness, self-mutilation, social isolation, laughing), depressive episodes, bipolar disorders, obsessive–compulsive disorders, infantile cholestatic icterus Lens dislocation, Marfan-like appearance, thromboembolic events, schizophrenia or psychotic episodes (rare), behavioural disorders, depression, obsessive–compulsive disorder, disorganized behaviour, delusions, depression, alteration of consciousness, peripheral neuropathy, coma, paraplegia, strokes, thromboembolic events	Infancy From childhood to adolescence Infancy to adulthood
Depression	Wilson’s disease Fabry’s disease (X-linked) Tay–Sachs/ Sandhoff disease or GM2 gangliosidosis Niemann–Pick type C Homocystinuria	Ophtalmology: Kayser-Fleisher rings mood disorders, behavioural and personality disorders, cognitive impairment, psychotic symptoms, dysarthria, anxiety Neural pain in hands and feet, cornea verticillata, hearing loss, stroke, renal dysfunction, proteinuria, asymmetric cardiac hypertrophy, angiokeratoma Speech and swallowing difficulties, unsteadiness of gait, spasticity, dystonia, cognitive decline, schizophrenia like psychosis, depression, mania, lower motor neuron disease, sensitive polyneuropathy, dysautonomia, spastic fright reaction, ophthalmology: cherry red spot, blindness Cfr supra Cfr supra	Adolescence and adulthood Adolescence and adulthood Infancy to adulthood Cfr supra Cfr supra

### Eating disorders

Concerning eating disorders and metabolic disorders only five relevant articles were found. Touati [61] describes frequent feeding disorders in children with methylmalonic and propionic acidurias, in which up to 60 % of patients needed a food supplement by tube. Deutsch et al. [62] report about a woman with anorexia nervosa and a mitochondrial myopathy, suggesting the possibility that the eating disorder was causally related to a more fundamental defect in mitochondrial oxidative metabolism. Symptoms of anorexia nervosa were also described in MELAS [63] and mitochondrial encephalomyopathy [64]. Sedel [3] mentions in his review that patients with a urea cycle disorder often experience protein intolerance and change their food habits becoming vegetarian or anorexic. Also, Gardeitchik [65] reports that protein aversion can be a diagnostic clue in patients presenting with food refusal, recurrent vomiting, behavioural problems, mental retardation and episodes of altered consciousness (see Table 6 for an overview).

**Table 6:** Possible underlying metabolic diseases in eating disorders

Possible underlying metabolic disease	Other symptoms and signs of the metabolic disease	Onset of the metabolic disease
Mitochondrial neurogastrointestinal encephalomyopathy (MNGIE)	Severe cachexia, gastrointestinal dysmotility, progressive external ophthalmoplegia, peripheral neuropathy	Infancy to adulthood
Mitochondrial encephalopathy lactic acidosis and stroke-like episodes (MELAS)	Mitochondrial encephalomyopathy, lactic acidosis, and stroke-like episodes	Infancy to adulthood
Methylmalonic and propionic aciduria	Developmental delay, cardiomyopathy, renal failure, opticus atrophy	From birth
Urea cycle disorders	Hepatomegaly, abnormal hair, hepatic fibrosis, intellectual and developmental disabilities, learning disabilities, confusion, delusion, seizure disorders, muscle tone change, reflex abnormalities, vomiting, headache, changing food habits	Infancy to adulthood

## Conclusions

The literature search concerning metabolic disorders and child psychiatric disorders was performed. Metabolic diseases represent rare but important causes of psychiatric diseases that remain isolated for years before more specific organic signs become obvious [3]. Psychiatrist should be aware of inborn errors of metabolism. In the literature, most relevant articles were found concerning ASD and psychosis in combination with a metabolic disorder. Based on the literature an overview for the different psychiatric disorders in the scoop of this paper was made in the combination with others signs and symptoms. This overview gives the child and adolescent psychiatrist some direction for further investigations and referral to a metabolic unit.

In the literature search, no guidelines were found when to look for a metabolic disease in a child presenting with a psychiatric disorder. A broad metabolic screening or routine metabolic screening carriers a low yield [8]. A metabolic work-up must be reserved for patients with clinical indicators of a metabolic disorder [8, 15, 66].

Further investigations are warranted in case of [3, 8, 15]:

- A positive family history of metabolic disease.
- Symptoms or signs are triggered by food intake (esp high protein content foods), fever, fasting, surgery (catabolism).
- Feeding difficulties, food refusal, failure to thrive, eating disorders combined with symptoms of myopathy or fatigue.
- Mental retardation and/or regression.
- Epilepsy, episodes of lethargy or confusion.
- Dysmorphic feature.

In most cases, there is a combination of neurological signs (epilepsy, ataxia, and catatonia), cognitive and motor dysfunction (hypotonia, hypertonia) and systemic signs of diverse organic involvement, cardiomyopathy, liver dysfunction, renal problems, immune deficiency, anaemia, and gastrointestinal problems (diarrhoea, obstipation, and pseudo-obstruction).

In this review, we did not include depression and anxiety disorders. The reason for this is that the focus of this review is on metabolic disorders as a comorbidity sharing similar pathogenesis, and a child psychiatric disorder being a clue to think about a metabolic disorder. In clinical practice, we see depression and anxiety after the diagnosis of a metabolic disorder, but rarely as a predictor of a metabolic disorder [1, 4]. Nevertheless, in the process of the literature search, we found a few metabolic diseases presenting with depressive episodes or anxiety such as Wilson disease and AIP [4] (see also Table 5).

This review is written from the perspective of a child and adolescent psychiatrist. The literature shows us that psychiatric diseases in adulthood can also reveal a metabolic disorder [3]. For instance, postpartum psychosis can be caused by a urea cycle disorder [67–69] and by GM2 gangliosidosis [70] and psychosis is also described in methylenetetrahydrofolate reductase deficiency (MTHFR) [71]. Therefore, knowledge of metabolic disease and their psychiatric manifestations is also warranted for adult psychiatrists. Finally, we hope to encourage the inclusion of inborn errors of metabolism in the differential diagnosis of psychiatric disease when appropriate as to allow and facilitate a prompt and correct diagnosis, followed by an effective treatment.

## Abbreviations

OXPHOS: Oxidative phosphorylation
OTCD: Ornithine transcarbamylase deficiency
3-OH-IVA: 3-hydroxyisovaleric acid
